# Vascular endothelial growth factor acts as an osteolytic factor in breast cancer metastases to bone

**DOI:** 10.1038/sj.bjc.6602417

**Published:** 2005-04-05

**Authors:** S E Aldridge, T W J Lennard, J R Williams, M A Birch

**Affiliations:** 1 The School of Surgical Sciences, The Medical School, Framlington Place, University of Newcastle upon Tyne NE2 4HH, UK

**Keywords:** VEGF, osteoclast, RAW 264.7, VEGFR1, VEGFR2, breast cancer

## Abstract

Vascular endothelial growth factor (VEGF) is a proangiogenic cytokine that is expressed highly in many solid tumours often correlating with a poor prognosis. In this study, we investigated the expression of VEGF and its receptors in bone metastases from primary human breast tumours and further characterised its effects on osteoclasts *in vitro*. Breast cancer metastases to bone were immunohistochemically stained for VEGF, its receptors VEGFR1 and 2 (vascular endothelial growth factor receptor 1 and 2), demonstrating that breast cancer metastases express VEGF strongly and that surrounding osteoclasts express both VEGFR1 and VEGFR2. RAW 264.7 cells (mouse monocyte cell line) and human peripheral blood mononuclear cells (PBMCs) were cultured with VEGF, RANKL and M-CSF. VEGF and RANKL together induced differentiation of multinucleated, tartrate-resistant acid phophatase (TRAP)-positive cells in similar numbers to M-CSF and RANKL. The PBMCs were also able to significantly stimulate resorption of mineralised matrix after treatment with M-CSF with RANKL and VEGF with RANKL. We have shown that VEGF in the presence of RANKL supports PBMC differentiation into osteoclast-like cells, able to resorb substrate. Vascular endothelial growth factor may therefore play a role in physiological bone resorption and in pathological situations. Consequently, VEGF signalling may be a therapeutic target for osteoclast inhibition in conditions such as tumour osteolysis.

Skeletal metastases are a common problem in the management of breast cancer patients. Many such patients suffer from bone pain and about one-third of patients with bone metastases develop one or more complications, for example, hypercalcaemia, pathological fractures or spinal cord compression (Coleman and Rubens, 1987). Metastatic breast cancer is incurable but its associated morbidity deserves attention. Understanding the processes involved in metastasis to bone could lead to optimal management of metastatic bone disease.

Bone is a unique environment as it is made up of a protein matrix that is calcified with crystals of hydroxyappatite. This means that the normal mechanisms that cancer cells use to create space in the extracellular matrix (ECM), such as the expression of matrix metalloproteinases (MMP), are ineffective. Therefore, breast cancer cells are believed to interact with surrounding cells to promote bone resorption and allow neoplastic invasion.

Vascular endothelial growth factor (VEGF) is an endothelial cell-specific mitogen (Veikkola and Alitalo, 1999). Binding of VEGF stimulates endothelial cells to degrade ECM, to migrate and to form tubes, through its interaction with two main membrane-bound cell receptors VEGFR1 and VEGFR2 (vascular endothelial growth factor receptor 1 and 2) (Ortega *et al*, 1999). Reduction in tissue oxygen partial pressure (*p*O_2_) causes VEGF mRNA transcription upregulation and stabilisation by osteoblasts (Akeno *et al*, 2001) along with some growth factors. Vascular endothelial growth factor may well not target endothelial cells exclusively as it has been shown to stimulate lymphocytes, granulocytes and monocytes, as well as stromal cells (Ortega *et al*, 1999).

Breast cancer cells have been shown to express VEGF and its receptors (Jong *et al*, 1998b). There is evidence to suggest that the selection and regulation of bony metastases is regulated by neovascularisation, with a switch from a relatively dormant metastasis into an ‘angiogenic’ phenotype leading to the production of tumour vasculature. This switch appears to be controlled by a balance of positive and negative angiogenic factors, particularly VEGF and angiostatin (Folkman, 1995).

Bone metastases from breast cancer tend to be osteolytic. Evidence supports the current notion that osteoclasts, induced either directly or indirectly by tumour cells, mediate osteolysis and that the growth factors released during bone resorption complete a vicious circle by stimulating tumour growth (Guise, 2000). Osteolytic bone tumours produce factors that regulate bone remodelling by altering rates of osteoclast and osteoblast differentiation and activity leading to a net increase in bone resorption (Jong *et al*, 1998a). Osteoclasts, the bone resorbing cells, are derived from haematopoietic stem cell precursors of the monocyte/macrophage cell lineage. *In vivo*, the precursors require cell to cell interaction with stromal cells expressing the cell membrane receptor, RANKL, in the presence of M-CSF, in order that they differentiate into osteoclasts (Yasuda *et al*, 1998). Circulating precursors in the mononuclear fraction of peripheral blood have been shown to differentiate into osteoclasts in the presence of soluble RANKL and M-CSF (Faust *et al*, 1999; Shalhoub *et al*, 1999). RANKL acts on osteoclast precursors via a membrane receptor, RANK (receptor activator of NF-*κ*B). Recently a non-RANKL-mediated pathway leading to osteoclastogenesis has been identified (Itonaga *et al*, 2004) There is growing evidence of a link between angiogenesis and bone turnover. Circulating precursors able to differentiate into endothelial cells are derived from the monocyte cell lineage, as are osteoclast precursors. Endothelial cells express similar phenotypic markers to osteoclasts, such as vitronectin receptor and calcitonin receptor. Recently there have been studies which show that antiangiogenic factors may inhibit osteoclast function and tumour osteolysis (Peyruchaud *et al*, 2003).

Human monocytes have been shown to express the receptors for VEGF, and VEGF is chemotactic for monocytes (Barleon *et al*, 1996). Recently, VEGFR1 has been shown to be expressed by osteoclasts in culture (Sawano *et al*, 2001). Vascular endothelial growth factor directly enhances osteoclastic bone resorption and survival of mature rabbit osteoclasts (Nakagawa *et al*, 2000). Niida *et al* (1999) found that rats, which did not produce M-CSF, were osteopetrotic as they had no osteoclasts, and that osteoclast induction could be restored by injection of VEGF. This study also showed that VEGF could substitute for M-CSF in cultures of rat bone marrow cells.

The aim of this study was to investigate the role of VEGF in the development of breast cancer metastases to bone. In this study, we have used immunohistochemistry to investigate the expression of VEGF and its receptors by human breast tumours infiltrating bone, and further characterised the effects of VEGF on osteoclastic development and activity *in vitro*.

## MATERIALS AND METHODS

### Clinical specimens

A total of 17 bone biopsies (12 excisional, five reamings) were obtained from the Department of Pathology, The Royal Victoria Infirmary, Newcastle upon Tyne. The biopsies had been taken from patients undergoing treatment at one of the Newcastle upon Tyne NHS Hospitals Trust's hospitals during the period 1993–2001. Patient ages at the time of biopsy ranged from 34 to 76 years. Sections of normal endometrium were used as positive controls for the VEGF and VEGFR1 and VEGFR2. Sections of a bone fracture were used as a positive control for osteoclasts staining for CD68.

### Immunohistochemistry

The specimens were stained with commercially available antibodies against VEGF and its receptors (VEGF, Flt-1 and KDR – Santa Cruz Biotechnology, Santa Cruz, CA, USA), and CD68 (DAKO, Denmark, DK-2600 Glostrup, Denmark), to identify osteoclasts. CD68 was chosen to identify osteoclasts as tartrate-resistant acid phosphatase (TRAP) staining was not possible on archived specimens which have been fixed in formalin and stored for extended periods of time. Alternative markers (such as vitronectin or calcitonin receptor) were evaluated as methods but these are markers also found on endothelial cells. Antigen retrieval was achieved with either microwave heat-induced antigen retrieval (VEGF and VEGF receptors) or trypsin (CD68).

After antigen retrieval, the slides underwent a protocol used to identify the appropriate antigens, modified from the ABC method initially described by Hsu *et al* (1981a, 1981b), following the protocol available from Vectorlabs (Vectorlabs, UK). The sections were blocked with serum of the same species as the biotinylated secondary antibody for 20 min. The sections were then incubated for the optimal time with primary antibody diluted in blocking serum at the optimal concentration in a humidified atmosphere.

Following the incubation with primary antibody, the samples were washed in PBS. The appropriate biotinylated secondary antibody was applied for 1 h and subsequently the specimens were incubated with streptavidin biotin complex (ABC) solution for 30 min.

The slides were exposed to 1 mg ml^−1^ diaminobenzidine tetrachloride (DAB – Sigma-Aldrich, Dorset, England, UK) chromogen for 5 min. The slides were then rinsed in running water and counterstained with Mayer's haematoxylin (BDH) (30 s) and Scott's Blue water. The slides were dehydrated and mounted. Cells that expressed the antigen would stain brown (DAB).

### Cell culture

#### RAW 264.7 cell line

The murine leukaemia virus-induced tumour monocyte cell line Raw 264.7 was obtained from the American Type Culture Collection (ATCC, Rockville, USA). This cell line is a monocyte/macrophage cell line, which has been shown in the past to differentiate into osteoclasts under stimulation with RANKL and M-CSF (Wei *et al*, 2001). Raw 264.7 cells were routinely cultured in 75 cm^2^ tissue culture flasks (Corning, Stone, UK) in DMEM culture medium at 37°C in an humidified atmosphere containing 5% CO_2_ in air. Cultures were maintained by subculture in the ratio 1 : 3 as required using a cell scraper. RAW 264.7 cells were cultured in modified Dulbecco's minimum essential medium (DMEM) (Sigma). In total, 10^4^ or 10^3^ cells were placed in eight-well chamber slides (Falcon) in 300 *μ*l DMEM and cultured in the presence of combinations of cytokines (rmVEGF (100 ng ml^−1^), rmM-CSF (30 ng ml^−1^), rmRANKL (100 ng ml^−1^)) for a period of 1 week with medium changes every other day.

#### Peripheral blood mononuclear cells (PBMCs)

The isolation of PBMCs requires the separation of whole blood by centrifugation through a density gradient (Boyum, 1968a, 1968b). Heparinised blood was obtained from healthy volunteers with informed consent. Whole blood was layered onto a sterile aqueous medium (Histopaque-1077 – Sigma) containing ficoll and sodium diatrizoate at a density of 1.077 g ml^−1^ at 25°C. This was then centrifuged at 500 **g** for 25 min at room temperature resulting in the separation of PBMCs at the blood/ficoll interface, the other white blood cells (WBC) and red blood cells (RBC) having passed through the interface. The PBMC interface was collected and washed with sterile phosphate-buffered saline (PBS) to remove any contaminating separation medium. The PBMCs were then pelleted by centrifugation at 500 **g** for 5 min. This pellet was then reconstituted in *α*-MEM culture medium.

5 × 10^5^ PBMCs were placed in eight-well chamber slides in 300 *μ*l *α-*MEM and cultured for 2 weeks in the presence of cytokine combinations (rhVEGF (100 ng ml^−1^), rhM-CSF (30 ng ml^−1^), rhRANKL (100 ng ml^−1^), Peprotech EC, London W6 8LL, UK). The medium was changed three times per week.

Following culture, the cells were fixed in 4% paraformaldehyde and stained for TRAP activity.

Each experiment was repeated three independent times

### Tartrate-resistant acid phosphatase staining

Osteoclasts express TRAP, an enzyme, which can be identified in cells through a histochemical staining methodology (Minkin, 1982; Allen *et al*, 1989). Staining solution (Sigma Diagnostics) was made up of acetate, naphthol AS-BI phosphoric acid and tartrate solution in 37°C water and fast garnet GBC salt was added to the solution. This solution was filtered and the slides were then incubated in the solution for 1 h at 37°C and then washed in water.

### Resorption assay

Her Majesty's Government Customs and Excise (UK) kindly donated confiscated ivory from illegal trade. The ivory was cut into 7 mm square slices 0.8 mm thick with a precision cutting saw (Isomet, Buehler) using diamond-tipped blade. The ivory slices were sterilised by soaking in 100% ethanol for 1 h and thoroughly washed and exposed for 30 min to ultraviolet light to each side of the square. The slices were then stored in sterile PBS before use.

Peripheral blood mononuclear cells were seeded down onto the ivory discs in 48-well plates at a density of 5 × 10^5^ cells in 300 *μ*l of *α*-MEM per well. The cells were then left to settle for 4 h before the medium was removed entirely and replaced with fresh medium and the relevant cytokines.

The cells were cultured on ivory for 3 weeks with medium changes every 3 days. Medium changes replaced two-thirds of the culture medium.

RAW 264.7 cells were seeded onto the ivory at a density of 10^4^ cells per well in 300 *μ*l of DMEM. The cells were left overnight, after which the cytokines were added. The cells were cultured for 2 weeks with medium changes three times per week.

In each individual experiment, treatment groups contained five separate ivory slices in identical wells. After culture, ivory slices were fixed in 4% paraformaldehyde and TRAP stained to identify osteoclasts.

The cells were then removed from the ivory through washing with 5% sodium hypochlorite and abrasion with cotton wool buds. Selected individual slices were initially imaged with scanning electron microscopy to identify resorption pits.

Once resorption had been identified, the slices were viewed under a reflected light microscope, used for metallurgic imaging. The images were captured as JPEG files and the area of resorption could be measured using image analysis (Image J). For quantification of each slice, 10 randomly chosen fields of view at 20 times magnification were captured. Thus for each treatment, 50 images were analysed (10 fields of view from five slices) giving a mean area of resorption.

### Statistical analysis

Results of the cell culture and resorption assay were processed with the statistical software Minitab 13 (Minitab Inc.).

## RESULTS

### Expression of VEGF and its receptors by breast cancer metastases to bone

All samples stained positively for VEGF and its receptors VEGFR1 and VEGFR2. The osteoclasts were identified as multinucleated cells associated with bone matrix, which stained positively for CD68 (a marker of monocyte macrophage cell lineage).

**Figure 1:**
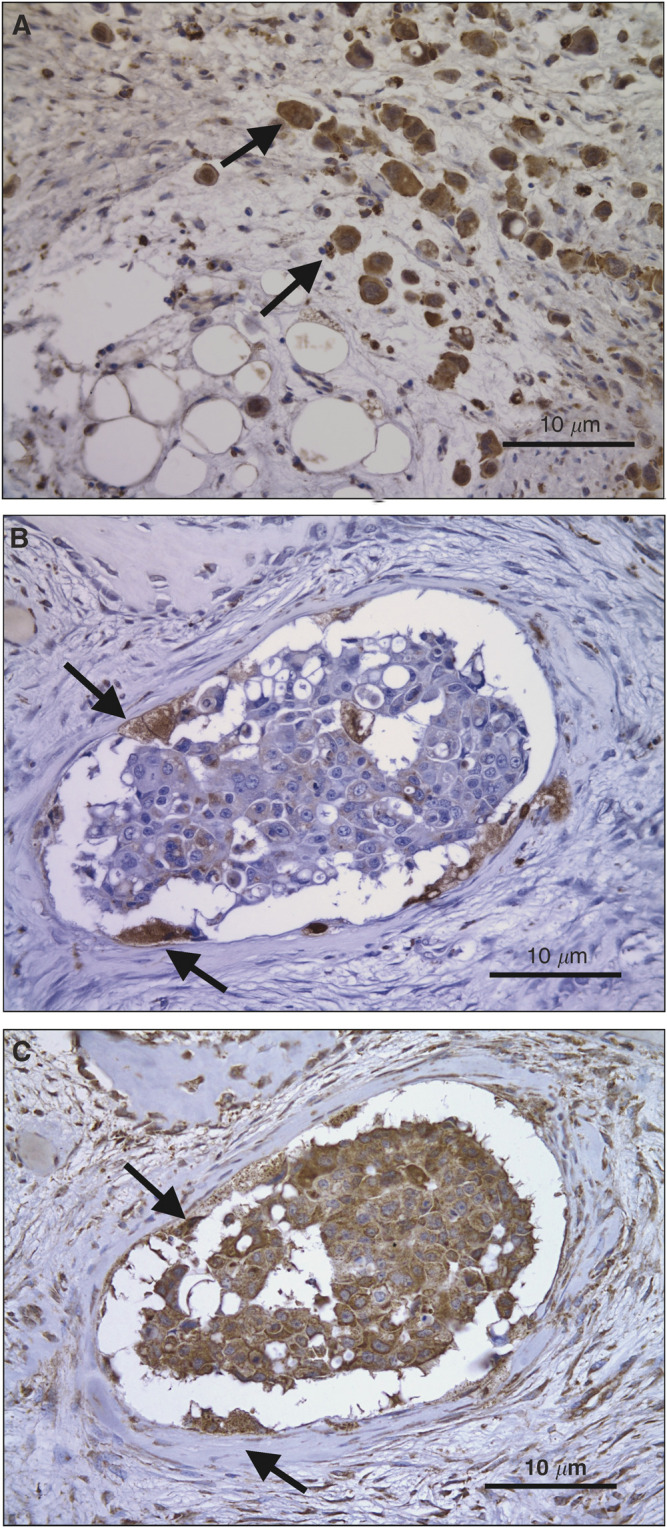
(**A**) Specimen stained with antibodies against VEGF. The large pleomorphic tumour cells (arrows) express VEGF at relatively high levels. Serial sections stained with antibodies against (**B**) CD68 and (**C**) VEGFR2. There is obvious expression of VEGFR2 by the central tumour cells. The osteoclasts express CD68 (arrows) and can also be seen in (**B**) to express VEGFR2.

Tumour cells were identified in the sections as those cells that were abnormal within the section, displaying pleomorphic features with hyperchromatic nuclei and high nuclear/cytoplasmic ratio. All sections of bone metastasis from breast cancer, stained with the antibody against VEGF, localised its expression to the tumour cells and some bone cells (Figure 1). All the tumour cells stained strongly and uniformly for VEGF implying high levels of expression. All sections stained for the receptor VEGFR1 with staining present in tumour cells and some of the bone cells. The tumour cells stained uniformly strongly and so there was no reason to count the cells. VEGFR2 antibody stained all of the tumour cells with no negative cells visible. Some of the bone cells stained for this receptor. The endometrium stained with the VEGF, VEGFR1 and VEGFR2 antibodies showed expression by the glandular structures as expected but the stroma was negative. The negative controls showed no staining.

### Osteoclasts express VEGF receptors

Osteoclasts were identified in 14 of the 17 sections stained with antibody to CD68. The three sections with no CD68 staining were full of tumour with no bone/tumour interface and hence had no osteoclasts present. In serial sections it was possible to identify osteoclasts that were present in more than one section. This allowed identification of the same osteoclasts, which had been stained by different antibodies in subsequent sections. Figure 1B and C shows serial sections staining for the VEGFR2 and CD68 antibodies. Table 1 shows the number of sections where osteoclasts were identified, and of those, the number of sections where osteoclasts were identified to express VEGF, VEGF receptors or CD68. Two specimens had osteoclasts, which did not express VEGFR1, but all specimens had osteoclasts expressing VEGF and VEGFR2 to varying degrees. These results are summarised in Table 1.

### Vascular endothelial growth factor supports differentiation of osteoclasts in the presence of RANKL

By culturing PBMCs in *α*MEM and in the presence of cytokines such as RANKL, M-CSF and VEGF, the cells acquire TRAP activity as demonstrated by the increased numbers of TRAP-positive cells after the culture period. These TRAP-positive cells can be divided into mononuclear TRAP-positive and multinucleated TRAP-positive cells. Mononuclear TRAP-positive cells may be cells on the pathway to a multinucleated phenotype. In this study, the osteoclast-like cells were defined as the multinucleated TRAP-positive cells, as described previously (Matsuzaki *et al*, 1998; Shalhoub *et al*, 1999).

**Figure 2:**
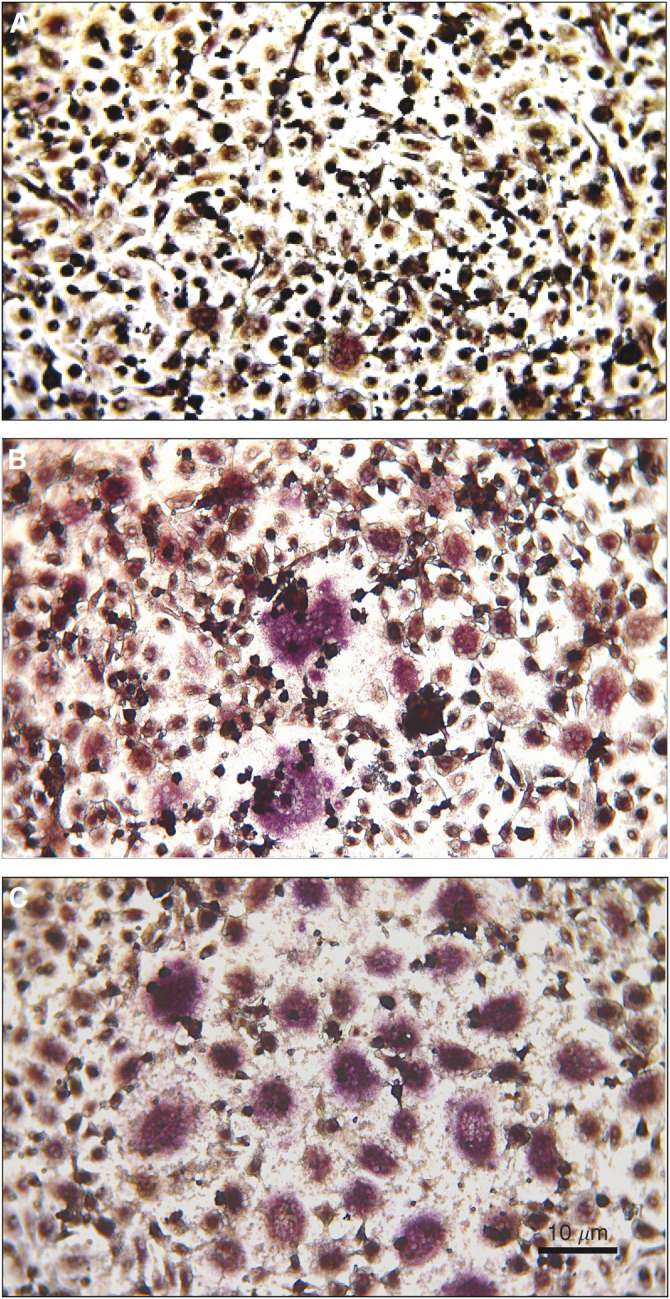
Tartrate-resistant acid phophatase staining of PBMCs after 2 weeks culture. (**A**) Negative control, (**B**) M-CSF and RANKL, (**C**) VEGF and RANKL.

**Figure 3:**
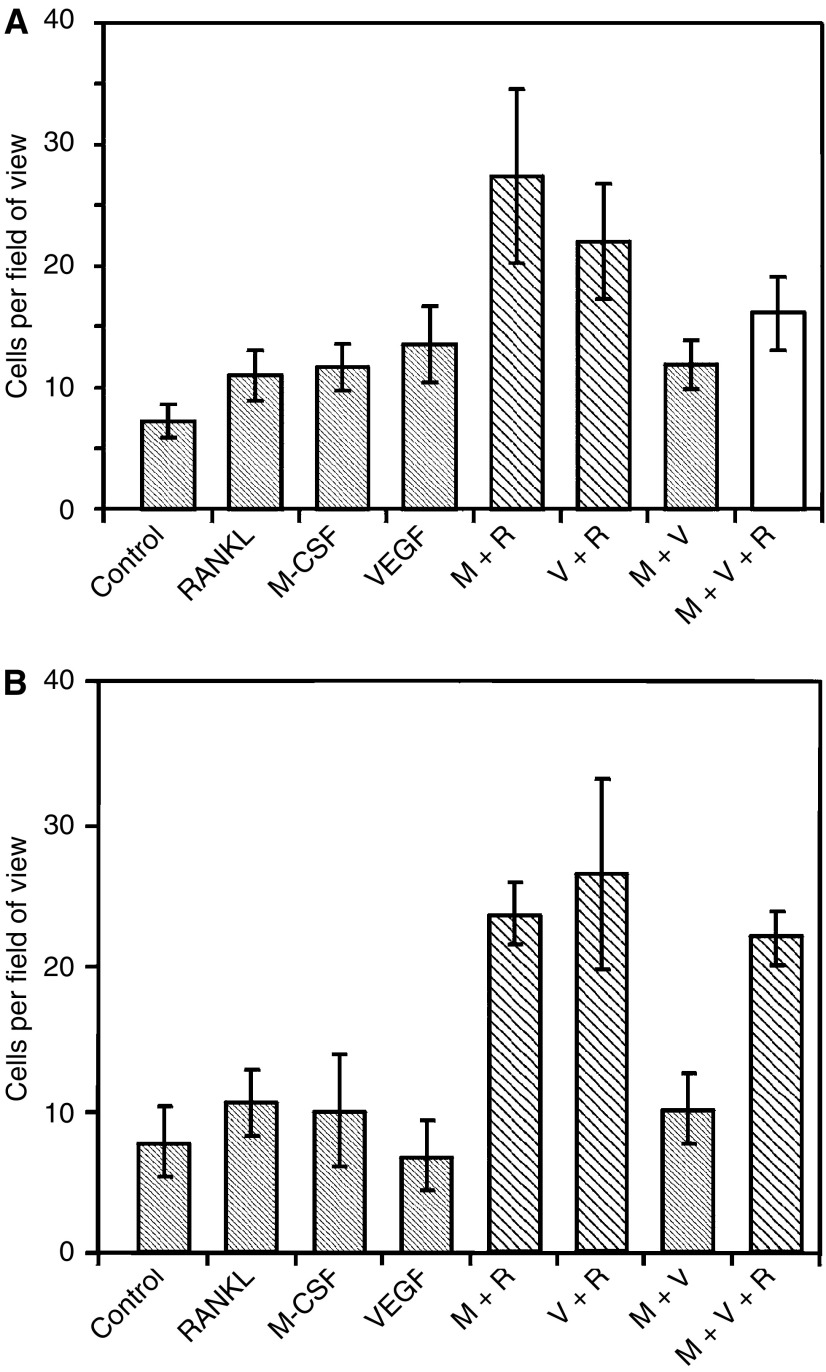
Graph showing mean numbers of multinucleated TRAP-positive cells after stimulation with cytokines (M=M-CSF, R=RANKL, V=VEGF). The experiments were carried out in both (**A**) PBMCs and (**B**) RAW 264.7 cells. The experiments were repeated in triplicate and typical graphs are shown here. Bars in stripes are significantly different on ANOVA at 95% CI from negative control. Grey bars are significantly different from positive control (M+R). White bars are different from both.

Culture of PBMCs for 2 weeks, while being exposed to combinations of the cytokines, RANKL, M-CSF and VEGF, resulted in the formation of multinucleated TRAP-positive cells (Figure 2). Stimulation of the cells with individual cytokines had little effect, whereas stimulation by M-CSF (30 ng ml^−1^) and RANKL (100 ng ml^−1^) or VEGF (100 ng ml^−1^) and RANKL induced differentiation of significant numbers of osteoclast-like cells, but there was no difference between these two groups. M-CSF and VEGF together did not increase the number of multinucleate TRAP-positive cells (Figure 3).

RAW 264.7 cells cultured for 1 week differentiated into multinucleated TRAP-positive cells when exposed to certain combinations of the cytokines, M-CSF (30 ng ml^−1^), VEGF (100 ng ml^−1^) and RANKL (100 ng ml^−1^). Incubation with M-CSF and RANKL or VEGF with RANKL resulted in significantly increased numbers of multinucleated TRAP-positive cells as compared to negative control (Figure 3).

The individual cytokines and M-CSF with VEGF had little effect on the numbers of osteoclast-like cells produced after culture. This is in agreement with the experiments on the PBMCs.

In these experiments, the numbers of cells that exhibit osteoclastic phenotype are less than the numbers in the PBMC cultures. This can be explained by the fact that there were fewer cells per well initially, but also these cells are an immortalised cell line that are being stimulated in an attempt to induce terminal differentiation. The TRAP positivity of these cells was noticeably reduced compared to the PBMCs and this may be explained by the same reason.

### Vascular endothelial growth factor stimulates osteoclasts to resorb substrate in the presence of RANKL

**Figure 4:**
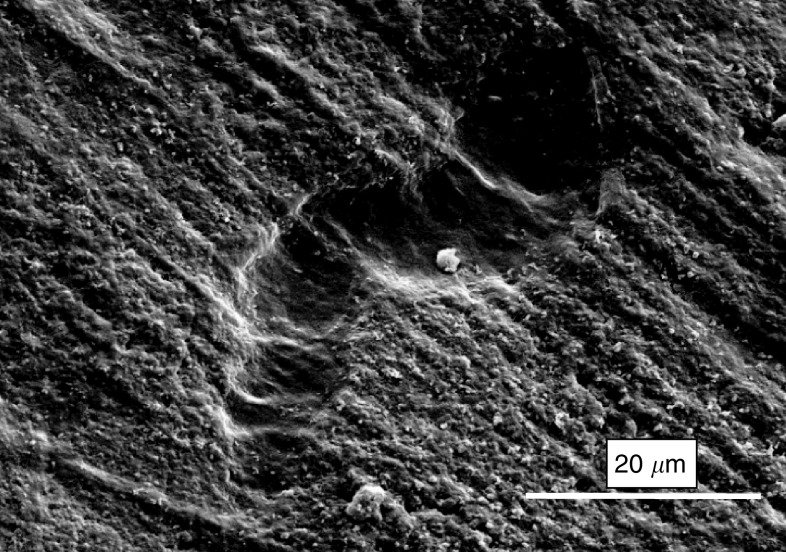
Scanning electron microscopy of ivory slice after culture with PBMCs stimulated by VEGF and RANKL. Example of resorption pit.

After 3 weeks of culture under appropriate conditions, the PBMCs on the ivory slices showed evidence of differentiation into multinucleated TRAP-positive cells (as expected from previous culture on eight-well chamber slides). After washing the cells off, the slices were examined by scanning electron microscopy after having been coated with gold (Figure 4). The resorption appeared as characteristic pits in the surface of the ivory. On imaging with a reflected light microscope, the areas of resorption could again be identified against the background of machining grooves.

**Figure 5:**
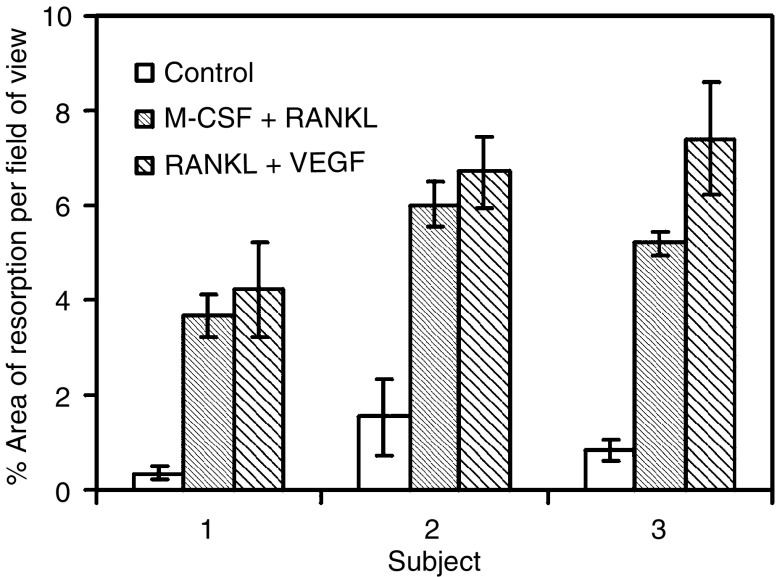
Graph showing the mean area of resorption after stimulating PBMCs for 3 weeks with the cytokines.

The area of resorption was estimated by delineating the resorption pits and then processing these images with the image analysis software. The cells attached to the ivory had been exposed to the cytokines M-CSF with RANKL and VEGF with RANKL and these treatments were compared to the negative control of no treatment. The results can be seen in Figure 5. The areas of resorption on the slices cultured in M-CSF and VEGF with RANKL were significantly increased as compared to the slices of the negative control. There was no significant difference between the two treatment groups. The cultures were repeated in triplicate and this result was born out in all three experiments.

The RAW 264.7 cells also differentiated into multinucleated TRAP-positive cells after culture, as had been seen in the chamber slide culture. However, the number of resorption pits seen on electron microscopy was markedly less than with the PBMCs. On the negative control ivory slices, there was no resorption seen. On the slices that had been exposed to M-CSF with RANKL and VEGF with RANKL, there were sparse areas of resorption. However, the number of pits were too few to make a meaningful count.

## DISCUSSION

Vascular endothelial growth factor has been shown to be an important factor in the progression of solid tumours. As any tumour grows it outstrips its blood supply and the tumour mass can become hypoxic. It is at this point that the tumour requires an ‘angiogenic switch’, where the tumour starts to produce factors to induce neovascularisation. VEGF has been shown to be one of the chief factors involved in the development of a tumour's blood supply. Several solid tumours have been shown to express VEGF and it has been identified as a factor, which conveys a worse prognosis. Levels of VEGF in the circulation of patients with cancer have been measured and again correlate with worse prognosis. Primary breast cancer has been studied by many research groups and VEGF is expressed more with increasing size and grade of tumour. The breast tumour cells were also seen to express the VEGF receptors and an autocrine role for VEGF on breast cancer cells has been hypothesised. In this study, we have shown not only that metastatic breast cancer cells in bone express relatively high levels of VEGF but also that they express VEGF receptors. Furthermore, this study revealed that osteoclasts associated with breast cancer metastases express both VEGF receptors. This is in keeping with the growing evidence available that VEGF has an action in skeletal biology. Monocytes, the precursors of osteoclasts, express VEGFR1 (Clauss *et al*, 1996) and there is some evidence that VEGFR2 is also expressed (Fernandez Pujol *et al*, 2000). This has been shown both at the protein level and the RNA level (Barleon *et al*, 1996). In mature osteoclasts, the presence of VEGF receptors has also been seen. VEGFR1 is seen on rat osteoclasts (Niida *et al*, 1999). Both VEGFR1 and VEGFR2 have been detected at the gene and protein levels in mature osteoclasts (Nakagawa *et al*, 2000).

Vascular endothelial growth factor has been shown to act on monocytes in various ways. Vascular endothelial growth factor is chemoattractant for monocytes (Barleon *et al*, 1996; Clauss *et al*, 1996; Sawano *et al*, 2001), and this seems to be mediated by the VEGFR1. Vascular endothelial growth factor also stimulates production of tissue factor (a procoagulant) by monocytes and endothelial cells and this is also mediated partly by VEGFR1 (Clauss *et al*, 1996). Interestingly, long-term stimulation of monocytes with VEGF induces the cells to differentiate into endothelial-like cells and express increasing levels of VEGF receptors (Fernandez Pujol *et al*, 2000). Taken with the previously reported data, our results show osteoclastic expression of VEGF receptors and suggest a role for VEGF in osteoclastic function.

There is growing evidence that VEGF can interact with monocytes, the precursors of osteoclasts, to induce differentiation into osteoclasts. Osteopetrotic mice, which lack M-CSF, have no osteoclasts. An injection of VEGF induces osteoclast recruitment in a manner similar to M-CSF injection (Niida *et al*, 1999). Vascular endothelial growth factor induces preosteoclasts from arthritic joints to differentiate, migrate and proliferate and this seems to be mediated by VEGFR1 (Matsumoto *et al*, 2002). Osteoclastic induction has also been shown to be increased around moving teeth when VEGF is administered locally (Kaku *et al*, 2001).

We have found in our study that VEGF, in the presence of RANKL can induce differentiation of osteoclasts from human monocyte precursors isolated from circulating blood from humans. The experiments have been repeated using the monocyte cell line, RAW 264.7, with similar results.

Following phenotypic markers allows the study of osteoclastogenesis but osteoclast function can only genuinely be assessed by assessing bone resorption. There is mounting evidence that VEGF has a role in osteoclast-induced bone resorption. Mature rabbit osteoclasts cultured on bone slices have increased numbers of resorption pits after exposure to VEGF (Nakagawa *et al*, 2000) and these osteoclasts express both VEGF receptors. The VEGF induced tyrosine phosphorylation of proteins in the osteoclasts. Nakagawa *et al* could not differentiate which receptor mediated this action. More recently, Henrikson has shown that activity of mouse osteoclasts can be increased by exposure to VEGF. This was done by measuring calcium release from metatarsals stimulated with VEGF and RANKL (Henriksen *et al*, 2003). The effect of VEGF was mediated by phosphorylation of ERK1/2 and this was shown to be necessary as inhibition reduced the effect of VEGF.

Our experiments have revealed that VEGF can substitute for M-CSF and stimulate the formation of osteoclasts. Vascular endothelial growth factor and RANKL together induced monocyte precursors to differentiate and resorb ivory in culture. Therefore, the localised production of VEGF, such as that observed in metastatic tissue, is likely to contribute to osteolysis in metastatic disease.

## Figures and Tables

**Table 1:** Expression of VEGF, VEGFR1 and VEGFR2 on osteoclasts present in breast metastases to bone

**Patient**	**Osteoclasts present**	**VEGF**	**VEGFR1**	**VEGFR2**
1	+	++	++	++
2	+	++	++	++
3	−	−	−	−
4	+	++	++	++
5	+	++	+	++
6	+	+++	0	+
7	+	+++	+	++
8	+	++	0	++
9	−	−	−	−
10	+	++	+	++
11	+	++	++	++
12	+	++	++	+
13	+	+++	++	++
14	+	++	++	+++
15	+	++	++	++
16	−	−	−	−
17	+	++	++	++

(−)=no osteoclasts present; (0)=negative staining; (+)=weak staining; (++)=moderate staining; (+++)=strong staining; VEGF=vascular endothelial growth factor; VEGFR1/2=vascular endothelial growth factor receptor 1/2.
